# A structured pathway for developing your complex abdominal hernia service: our York pathway

**DOI:** 10.1007/s10029-020-02354-9

**Published:** 2021-02-18

**Authors:** O. Smith, T. MacLeod, P. Lim, P. Chitsabesan, S. Chintapatla

**Affiliations:** 1 York Abdominal Wall Unit, Department of General Surgery, York Teaching Hospital NHS Foundation Trust, Wigginton Road, York, YO31 8HE UK; 2 Department of Plastic Surgery, York Teaching Hospital NHS Foundation Trust, Wigginton Road, York, YO31 8HE UK

**Keywords:** Abdominal wall hernia, Abdominal wall Reconstruction, Pathway, Protocol

## Abstract

**Purpose:**

Clinical pathways are widely prevalent in health care and may be associated with increased clinical efficacy, improved patient care, streamlining of services, while providing clarity on patient management. Such pathways are well established in several branches of healthcare services but, to the authors’ knowledge, not in complex abdominal wall reconstruction (CAWR). A stepwise, structured and comprehensive approach to managing complex abdominal wall hernia (CAWH) patients, which has been successfully implemented in our practice, is presented.

**Methods:**

A literature search of common databases including Embase® and MEDLINE® for CAWH pathways identified no comprehensive pathway. We therefore undertook a reiterative process to develop the York Abdominal Wall Unit (YAWU) through examination of current evidence and logic to produce a pragmatic redesign of our own pathway. Having introduced our pathway, we then performed a retrospective analysis of the complexity and number of abdominal wall cases performed in our trust over time.

**Results:**

We describe our pathway and demonstrate that the percentage of cases and their complexity, as defined by the VHWG classification, have increased over time in York Abdominal Wall Unit.

**Conclusion:**

A structured pathway for complex abdominal wall hernia service is one way to improve patient experience and streamline services. The relevance of pathways for the hernia surgeon is discussed alongside this pathway. This may provide a useful guide to those wishing to establish similar personalised pathways within their own units and allow them to expand their service.

**Electronic supplementary material:**

The online version of this article (10.1007/s10029-020-02354-9) contains supplementary material, which is available to authorized users.

## Introduction

Clinical pathways may be associated with clinical efficacy and improved patient safety [1]. They are a method of “standardising the progression of treatment, to support patient care and facilitate clinical decision making” [2]. Clinical pathways streamline patient services in a health system that is overburdened and facing fiscal challenges [3]. Such pathways are well established in several healthcare services such as perioperative medicine [3]. To our knowledge, no such pathway has been published for complex abdominal wall hernia (CAWH) patients.

The incidence of CAWH is increasing and represents a significant disease burden [4–6]. General and plastic surgeons that operate on such patients typically do so over and above other clinical commitments and parallel cancer workloads; this means that the time to manage these complex patients is at a premium. Complex abdominal wall reconstruction (CAWR) is an emerging subspecialty and presently there is little by way of a structured multi-disciplinary team agreement to standardise the management of these complex patients [7]. A structured CAWH patient management pathway may be one way to mitigate some of the challenges in managing these patients.

In York, complex abdominal wall surgery had been historically performed in isolation by general surgeons with a modest effect. Most cases were Ventral Hernia Working Group (VHWG) grade 1–2. As a surgical group, we felt we had to cater for all types of hernias (VHWG grade 1–4) and offer this extended service locally. This required a process of radical redesign.

We started with a background search of Medline and EMBASE for pathways or protocols for the management of complex abdominal wall hernias. This was performed by an independent expert in database enquiries and literature reviews. The free-text search string on both databases was (“complex abdominal” OR “abdominal wall” OR ventral OR incisional) ADJ hernia* including thesaurus terms on both Medline and Embase. The Cochrane Database of Systematic Reviews, Epistemonikos, the Centre for Reviews & Dissemination, TRIP database, NHS Evidence, PubMed, UpToDate, BMJ Best Practice, medRxiv and Google Advanced Search for additional information were reviewed. There were no language or date limits on the search. This yielded 60 papers of which 3 were relevant [8–10]. None described a complete pathway.

We designed a local pathway for these patients in our organisation, underpinned by governance, that aimed to be both practical and efficient. It is hoped that this will serve as a potential framework for others wishing to establish their own pathways within their units in a safe manner.

## Our York pathway

**Fig. 1:**
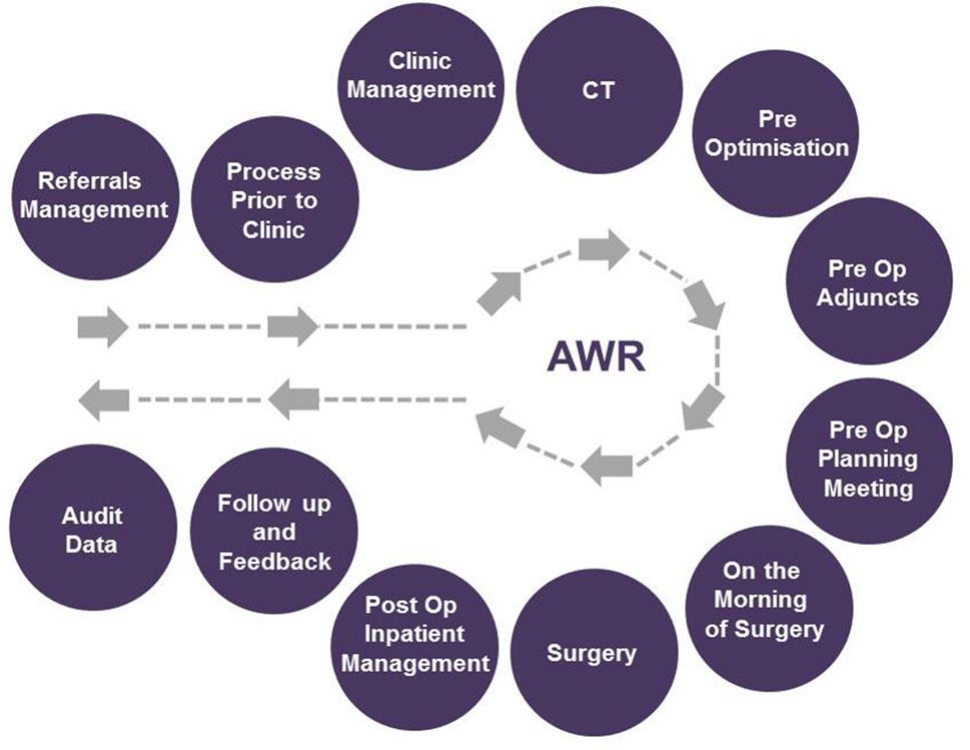
York CAWH Pathway

The York CAWH Pathway (Fig. 1) was designed around the current best evidence and guidelines pertaining to the management of such complex patients [11].

The patient journey begins at the initial referral and then moves through each stage of the pathway as summarised in Fig. 1. While respecting the principles of individualised care, all patients referred to the CAWR team broadly go through the same pathway. Further, even though the pathway is presented sequentially as a number of steps, the authors acknowledge that certain steps may occur concurrently. Progress of a patient through the pathway is constantly monitored. The patients are tracked by a specialist dietician on a regular basis. Once they approach the ideal weight, departmental administration staff will arrange a clinic review. The waiting list staff coordinate efforts so that pre-operative adjuncts such as intramuscular botulinum toxin injections can be scheduled and a planned surgical date organised. The administration team coordinate the availability of surgeons, clinic review, pre-assessment and appropriate post-operative level care such as HDU beds. The pathway has 12 distinct stages.

1. *Referrals management*—this is an established process for receiving referrals. It provides clarity concerning which consultant receives the referral, how it is logged and is audited. Maintaining a referral database and referral template manages the system optimally [12].
2. *Process prior to clinic*—we send:

- An information leaflet on how the CAWH clinic runs
- An information leaflet and a consent form for photography
- A ten-page health questionnaire (see Supplementary file) including the VHWG factors, comorbidities, and surgical history such as previous operation, operative outcome, surgical site infection (SSI), mesh used and whether it had been explanted. This saves time in the clinic appointment and is useful since much information has not yet been digitised.

3. Clinic management

The York Abdominal Wall Unit has set up a combined clinic with a GI surgeon and a plastic surgeon.

- Patients are assigned a 30-min appointment. This involves reviewing documentation and health questionnaire (see Supplementary file). Supplementary information is collected with regards to operative details, which mesh was used, the planes disrupted. We promote patient ownership of their condition [13] and increased patient understanding of the factors that adversely impact it [10]. The resultant effect is increased motivation to self-influence modifiable factors, e.g. smoking and weight loss [14].
- The hernia footprint as per the European Hernia Society classification and VHWG grade [15, 16] are recorded. The Carolina’s Equation for Determining Associated Risks (CeDAR) score is calculated with the patient [17]. Modifiable factors, such as smoking, are adjusted within the CeDAR algorithm to visually reduce the percentage complication rate in front of the patient. By engaging in such practices, patients see how the VHWG grade is modified and appreciate that a shift from grade 2 to 1 could translate to a risk shift from 29 to 14% [16, 18]. This, coupled with calculating optimal target weight loss, promotes behaviour modification in a pragmatic manner.
- A specialised nutritionist clinic runs a parallel appointment. They measure the following parameters: BMI, grip strength and fat-free mass (using Bioimpedance) [19]. A detailed dietary plan is agreed, information leaflets are provided and a time frame is agreed for review. A follow-up plan which monitors weight loss but preserves physical strength is implemented.
- Protocoled patient photographs are taken in the clinical photography suite. A video is taken with the patient performing a straight leg raise to allow dynamic assessment of the inter-rectus gap under tension. The photographs and videos (with the consent form for photography) are stored and accessed within the framework of Information Governance of the Hospital.
- Management decisions are taken in the clinic as to whether a CT scan, colonoscopy or prehabilitation services are needed.
- If appropriate, the patient is placed on a surgical list and a provisional operative plan is created which includes the type of mesh to be used, the anatomical plane in which the mesh will be placed, whether explantation of mesh would be performed, the time allocation for the case and whether component separation may be needed (see Supplementary file).
- Finally, a structured letter with pre-formatted headings is dictated to the referring doctor (see Supplementary file). Procedure-specific paragraphs (PSPs) are used and serve to provide standardised content. The procedure-specific paragraph outlines risks and benefits of the CAWR. A pre-formatted letter ensures that all relevant details are uniformly documented and yet personalised. A copy of the letter is sent to the patient with an outline of the proposed plan.
- Patient information leaflets (PILs) are provided on

1. CAWH and its management,
2. Recovery after major abdominal surgery including physiotherapy post-operatively and
3. Post-discharge information (Fig. 2).

**Fig. 2:**
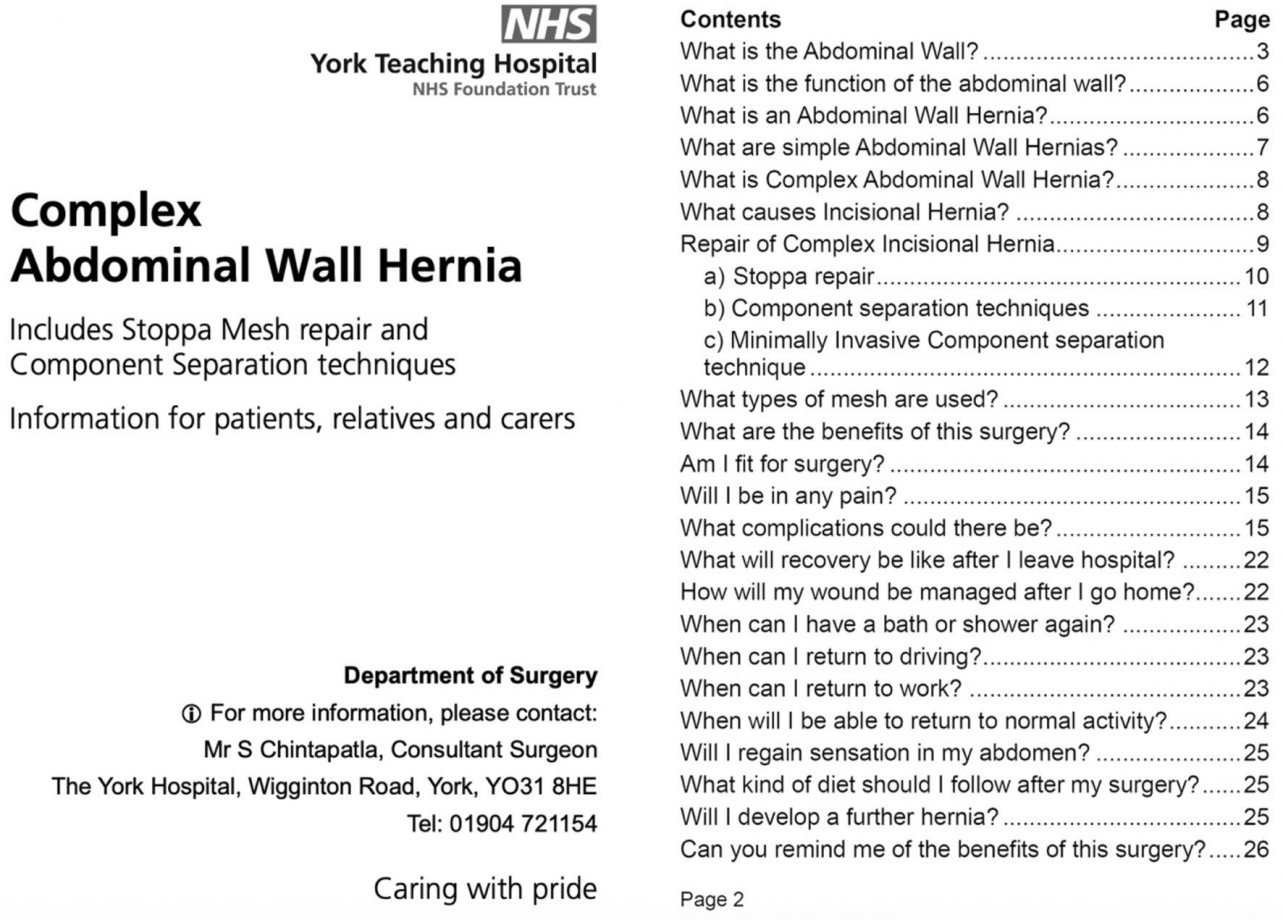
PIL about CAWH surgery
4. CT scan

A recent CT scan is necessary within the last year. It allows examination of: inter-rectus distance, multiplicity of defects, the oblique muscles (including their bulk), previous component separation and mesh placement, GI fistula (if present), sarcopenia and other incidental findings [20].

Radiologists provide hernia sac volume and the relative abdominal compartment volumes using Tanaka et al. [18], or Sabbagh et al. [19] calculations. These help in planning adjuncts to surgery such as pre-operative botulinum toxin or progressive pneumoperitoneum (PPP) [21, 22].

5. Pre-optimisation

By the time the patient comes to this stage in the pathway, programmes like smoking cessation, weight management, diabetes optimisation and nutritional support would have already been established and tailored to patient need and community availability [11]. The patient then undergoes further optimisation with pre-operative protocols to normalise haemoglobin levels, managing medications for comorbidities, and consideration of coincidental immunologics [11]. We assess frailty (Clinical Frailty Scale) and use strategies to improve strength and endurance [23]. Cognitive testing is part of perioperative assessment. In our unit, we also train patients on how to effectively use pre-operative incentive spirometry (to reduce post-operative pulmonary complications) and those strategies described by WHO to reduce post-operative surgical site infections [24].

As part of pre-optimisation, we consider whether bariatric multi disciplinary team (MDT) input is needed to answer the question of optimising diabetes and endocrine (e.g. thyroid) management. Access to bariatric MDT allows the involvement of further services, e.g. specialist dietician support, counselling services, weight management and specialist bariatric surgeon support with regards to need for bariatric surgery prior to/along with/after a massive complex abdominal wall hernia and the type of bariatric surgery intended.

6. Pre-operative adjuncts

The aim of pre-operative adjuncts is to complement processes that:

- Appose the recti abdominis without tension
- Return the hernia contents into the abdomen in a phased manner to reduce the incidence of post-operative intra-abdominal hypertension and compartment syndrome.

An assessment as to whether further adjuncts are required is based on an assessment of whether the goals are achievable. The two adjuncts to be considered are:

1. Botulinum toxin injection (also called chemical component separation) [25, 26]: A musculoskeletal radiologist administers a specific botulinum toxin Dysport® type A under ultrasound guidance 3–4 weeks prior to the procedure. In general, 300 units is administered on each side of lateral abdominal wall (with the dose distributed approximately 50 units in each muscle medially and 50 units laterally at each site making a total of 100 units in each of the three muscle layers). We aim to titrate the dose according to relative muscle bulk. In our experience the bulk of external oblique muscle is greater than the internal oblique muscle, which is greater than the transverse abdominis muscle, but acknoweldge that anatomy varies.
2. Progressive Pneumo Peritoneum (PPP) [25, 27]: if indicated PPP needs careful multi-disciplinary team engagement. Collaboration with the development of governance systems is the key to safe delivery. Our assessment includes cardio-pulmonary exercise testing, careful venous thrombo embolism (VTE) assessment (which may lead to placement of an IVC filter) and monitoring of both renal and lung functions. Our protocol involves insufflation of 500mls–1000mls carbon dioxide on alternate days, with a gradual increase in volumes, the end insufflation point being when the patient reports pain. Presently, there is no consensus regarding exact volumes with which to insufflate [28].

7. Pre-operative planning meeting

The aim of surgery is to:

- Appose the medial borders of the two recti abdominis muscles
- Return the hernia contents into the abdomen without undue tension
- Reconstruct the abdominal wall anatomy
- Restore abdominal wall form and function
- Adopt techniques to minimise SSI and hence potential recurrence

A consultant gastrointestinal (GI) surgeon and consultant plastic surgeon appraise the provisional surgical plan formulated in clinic, which includes clinical photographs, radiological investigations and tabulated physiological information including bariatric MDT discussions (if relevant).

Under this guidance, several decisions are made:

1. Types of skin incision (e.g. midline, elliptical, Fleur de Lis, etc.)
2. Types of mesh (e.g. synthetic, bioresorbable, biological [29])
3. Anatomical plane for mesh insertion [30]
4. Whether a previous mesh needs explanting
5. Whether concomitant GI surgery (e.g. GI fistula, reversal of stoma, restoration of GI continuity or bariatric surgery) is required
6. Whether component separation is required [31, 32]

There is a strong focus on encouraging trainees in this field within our unit, and the pre-operative planning meeting is an excellent time to teach and discuss operative principles and their application.

The above processes are reiterated in a further patient appointment with a pre-assessment nurse.

8. Morning of surgery checks

The value of this meeting is to review the operative plan and address any patient issues raised.

The process involves re-examining the patient, reviewing the definitive operative plan, marking the incision and planned skin resection, additional clinical photographs, and consenting for theatre [33]. Our consent form mirrors the procedure-specific paragraph in the clinic letter, the PIL written in Plain English previously provided and this iterative process reinforces the concepts of risk and benefit of CAWR [33]. Appropriate pre-assessment nursing checks are performed with documentation of consent on a pre-printed consent form which we have published before [33].

9. Surgery

**Fig. 3:**
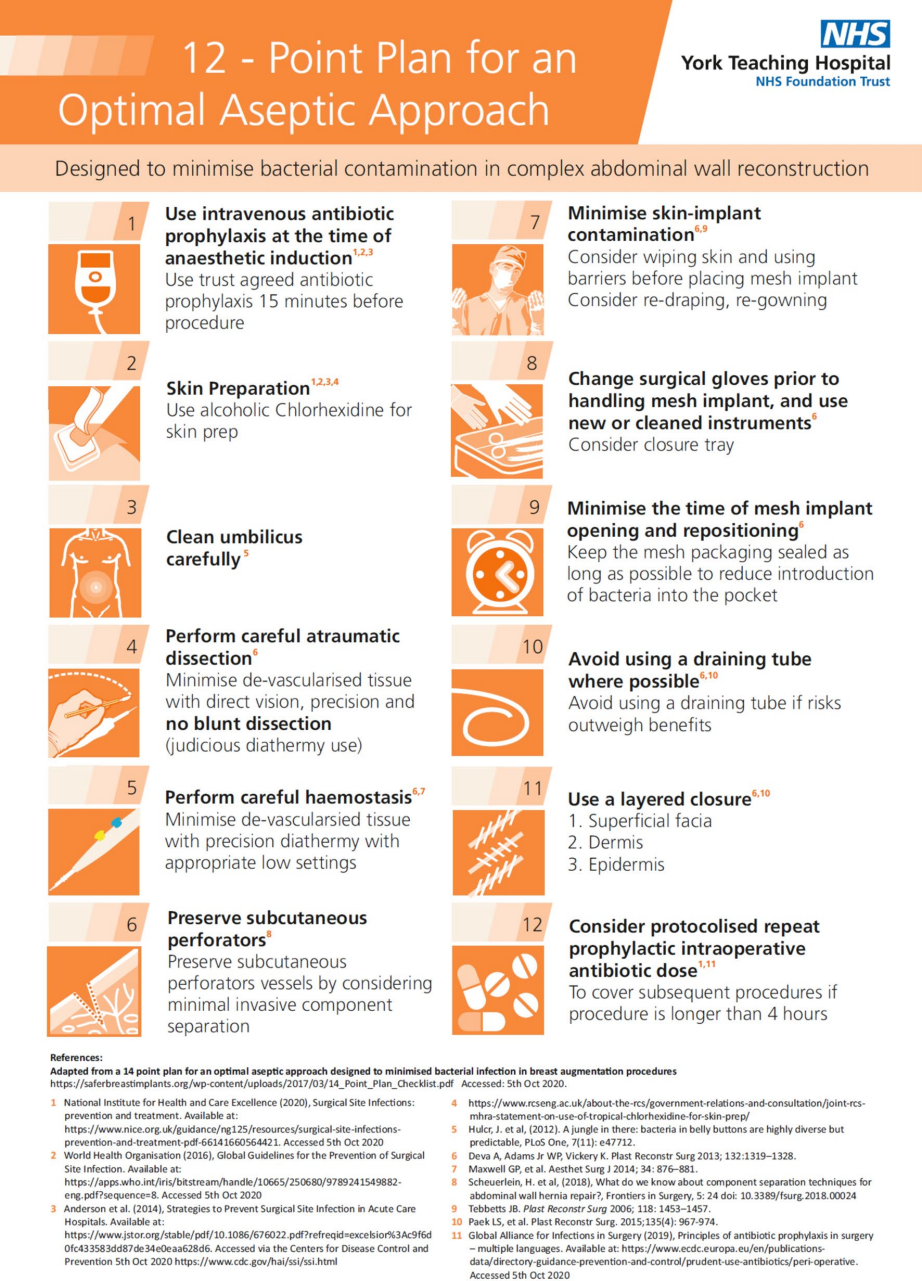
The 12-point plan to minimise surgical site infection (SSI) in CAWR

After a WHO briefing followed by a general anaesthetic, standard surgical checks and procedural steps are followed. The surgical team use a 12-point plan (Fig. 3) based on a 14-point plan published by breast surgeons in 2013 [34] designed to minimise bacterial contamination of breast implants at the time of surgery [35]. The 12-point plan incorporates evidence-based strategies into a logical stepwise CAWR checklist. The aim of the plan is to reduce the number of bacteria that may contaminate the CAWR mesh implant.

The 12-point plan is another way of improving organisation and therefore safety in a high-risk area [35]. The use of checklists in surgery has gained traction following an abundance of research in medicine and other fields [36–38]. Additionally, negative pressure wound dressings may be used, and the implant registry requirements are fulfilled [39].

10. Post-operative management

Particular care is taken to build nursing teams that deliver protocol-led care of these complex patients including drain management, general physiology and an abdominal wall support binder. Physiotherapists are involved in encouraging incentive spirometry and early mobilisation in keeping with enhanced recovery principles [40].

11. Follow-up and feedback

Patients are discharged with a 3-month follow-up appointment whereupon the wound is reviewed and further photographs are taken. A letter detailing the outcomes, further management is sent to the referring clinician. An email is sent to the referring clinician with pre- and post-operative images demonstrating the outcomes.

We evaluate “return to form and function” and “*quality* *of life*” [41]. In pandemic situations and for patient comfort, this aspect of clinic may be performed using video consultations. We use the application Attend Anywhere® (www.anywhere.com) in our unit.

12. Audit data

All elements of the York Pathway are audited. This is critical for service evaluation and constant quality improvement [42].

## How has this pathway improved our service?: our experience

The aim of this redesign was to allow the surgical group to tackle more complex hernias, whereas previously we were treating these conservatively. Since introducing elements of the pathway in 2014, it has allowed the management of more complex cases, i.e. more VHWG Grade 3 and 4 cases.

**Fig. 4:**
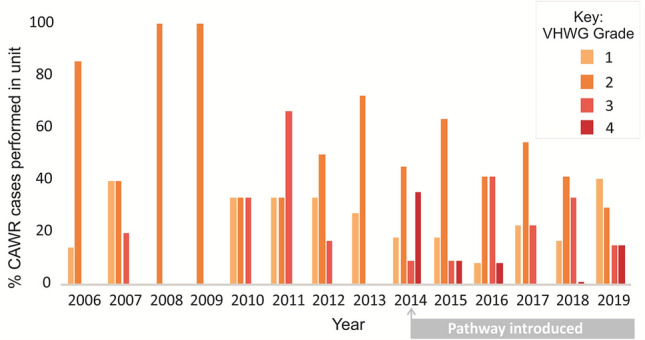
Graph illustrating the percentage of overall cases that were VHWG grade 1, 2, 3 or 4, over time

The percentage of cases and their complexity, as defined by the VHWG classification, has increased over time in the York unit (Fig. 4). As a percentage of overall VHWG grade 3 and 4 cases, 23% were performed pre-pathway compared with 76% post-pathway implementation. 2020 data has not been included owing to COVID-19 limiting access to elective surgeries.

## Discussion

There are several reasons why pathways may be important for the CAWH surgeon. Clinical pathways have been shown to be linked to improved patient safety [1–3]. Structure, in the form of a pathway or guideline, improves patient outcomes and reduces cost [43]. Well designed pathways, which involve all the interested stakeholders in their design, help to assimilate clarity, continuity, competence and efficiency both for the patient and primary and secondary care [1, 2]. They may streamline services by providing both physical and environmental organisations, ensuring that each individual patient has the right interventions performed at the right times by the right professionals [2].

Schneider et al. [44] demonstrated that pathways limit workplace distractions and aid memory recall via distilling a complicated management plan into something easily digestible and implementable in the correct sequence. Something ad hoc is difficult to follow and inconsistent. Pathways may provide a clear format that identifies what needs to be done for patients and when. Without a pathway, patient progress may be stilted. Healthcare professionals are faced with an unprecedented amount of information to retain and recall. This may lead one to question how team members remember all aspects of the York Pathway. The 12 steps of the pathway are logically ordered from referral to management. They run from introduction to initial assessment and initiation of prehabilitiation to pre-, peri- and post-operative management. This is not an unusual process and is applied throughout surgical specialties. We have developed a logical and simple pathway, which has complex management decisions integrated into it such that it has become routine with progression at one level, ensuring that the next step is evaluated/managed.

It is a human mechanism to forget. The 12 stages used in the pathway result in a form of “cognitive economy”, i.e. they are “hooks” that reduce cognitive effort required in recall [45]. If systems are too overloaded and illogical, it would be impossible to keep track of everything all of the time. That is the human condition. Our York pathway has twelve stages which are logically ordered and connected to one another and which are not all exclusive to CAWH patient, e.g. pre-operative planning and surgical checks, to aid team recall. In our experience, it is not too cognitively taxing. Furthermore, repeated use leads to familiarity and automation, and easy access to the pathway and related materials via the hospital intranet are also beneficial.

Surgery is a high-risk speciality and inconsistencies in patient care and a lack of forward planning have potential disastrous effects [46]. Forward planning and a structured, evidence-based approach to CAWH patient care via a pathway may help [2, 11, 25–27]. Beyond this, well-organised pathways provide stability. Hospital teams vary constantly, and individuals within a team have different strengths [47]. Providing structure breeds familiarity, repetition, excellence and a base line of what is expected for new staff. Such known patterns reduce uncertainty and improve productivity [2].

There is an argument as to whether a pathway is needed or whether a checklist would be more beneficial. It is important to be cognisant of the distinctions between a checklist and a clinical care pathway such as this. Checklists are lists of actions that must be performed in a specific setting, e.g. the intra-operative 12-point checklist designed to minimise the risk of infection in CAWR (Fig. 8) [48]. Checklists are listed algorithms designed to walk users through specific tasks aiming to reduce error. Commonly, errors are the result of either a lack of knowledge or failure to implement knowledge and even the most experiences healthcare professional can make an error. They complement and are built into more complex clinical pathways. Conversely, clinical pathways, like the one presented here, are a systematic way to transfer current best practice between users in the same or different institutions, thus allowing appropriate care and follow up for specific patient groups [49].

Within our hospital, several other pathways for different conditions are already in place and are associated with positive patient outcomes, e.g. the York Perioperative Model for Pre-assessment (details may be found here: https://www.yorkperioperativemedicine.nhs.uk/health-professionals/the-york-model/pathway-documents/?access=1). Equally, pathways are not new. A good and familiar example is the advent of integrated cancer care pathways [50]. We use the same concepts and apply it to CAWR.

We feel that what we have presented here is a logical pathway that is patient-centred and evidence-based to manage CAWH patients.

## Conclusion

Challenges to overcome the vicissitudes of CAWH practice require creative solutions. We have adjusted to face the challenge posed by increased CAWH service demand within our unit. We have developed an organised pathway that streamlines the CAWH service and has allowed us to manage more complex CAWH cases surgically. Here we present our comprehensive pathway that has been implemented successfully in our practice. We appreciate that this “York pathway” is felt to work well within our centre but perhaps may not work for others depending on service availability, needs of the unit and the country in which one is practicing. Despite this, we believe that a structured approach to CAWH and the unification of services may be one way to improve patient experience, outcomes and streamline services. We hope that our experience, and the blueprint of our service outlined here, may serve as an adaptable guide to others who wish to establish their own pathways within their units.

## Electronic supplementary material

Below is the link to the electronic supplementary material.

Supplementary file 1 (DOCX 494 kb)

## Data Availability

Not applicable. Not applicable.
