# Supplementary material for: A structured pathway for developing your complex abdominal hernia service: our York pathway
Source: Hernia. 2021 Feb 18;25(2):267–75. doi: 10.1007/s10029-020-02354-9 (PMC7890783; doi:10.1007/s10029-020-02354-9)
Supplement: Supplementary file 1 — (DOCX 494 kb) [file 10029_2020_2354_MOESM1_ESM.docx]

Hospital Ref: «CASENOTE»

NHS Number: «NHS_NUMBER»

«LETTER_DATE»

| **Private & Confidential**  «TITLE» «FORENAME» «SURNAME»  «ADDRESS_LINE_1»  «ADDRESS_LINE_2»  «ADDRESS_LINE_3»  «ADDRESS_LINE_4»  «TOWN»  «POSTCODE» | **«MAIN_LOCATION_ADDRESS»**  **Outpatient Enquiries Open 8am – 5pm**  **Monday to Friday**  **Telephone: (01904) 726400**  **To request to reschedule or cancel, visit**  www.yorkhospitals.nhs.uk/appointment |
| --- | --- |

Dear «TITLE» «SURNAME»

We have arranged an appointment for you to come to the Complex Abdominal Wall Hernia Clinic at the Outpatient Department at «MAIN_LOCATION».

Your appointment is on «APPOINTMENT_DATE» at «APPOINTMENT_TIME»

Please complete the attached questionnaire and consent form and bring along with you to your appointment. The overall appointment could take 3-4 hours and will include photography and other speciality consultations.

If you have an abdominal wall stoma, please bring a change of stoma bag with you as this may need to be removed during clinical examination and for clinical photographs.

Your appointment will be in the Main Outpatient Department. To get to the Outpatient Department come into the hospital at the main entrance and the Outpatient’s reception will be in front of you, to your left. Please tell the receptionist that you have arrived.

If you require hospital transport to bring you to your appointment, please contact the Patient Transport Service on «PATIENT_TRANSPORT_NUMBER» You will be asked some questions to find out about your transport needs and assess your eligibility, and will need the details of your appointment on this letter. Eligibility is based on medical need and therefore not all patients will be eligible for this service.

For more information on transport links to York Teaching Hospital NHS Foundation Trust sites please visit the link [www.yorkhospitals.nhs.uk/travel](http://www.yorkhospitals.nhs.uk/travel).

Your appointment with us is important and you should always try to keep it. To request to reschedule or cancel your appointment, you can complete our short form by visiting www.yorkhospitals.nhs.uk/appointment or call the Contact Centre on **(01904) 726400**. Please note that if you cancel your appointment on two consecutive occasions; or if you fail to attend your appointment without letting us know, we will discharge you back to the care of your GP.

Yours sincerely

Outpatient Appointments

**Private & Confidential**

«TITLE» «FORENAME» «SURNAME»

«ADDRESS_LINE_1»

«ADDRESS_LINE_2»

«ADDRESS_LINE_3»

«ADDRESS_LINE_4»

«TOWN»

«POSTCODE»

**Abdominal Wall Reconstruction - Health Screening Questionnaire**

**Date: …………………..**

We would be very grateful if you could complete the following questionnaire. The questionnaire is designed to give us an overview of your hernia and your general health which will help us in ensuring that you are optimally prepared for any surgery.

**Clinical Photographs:**

As part of the surgical planning process we normally take measurements of the hernia together with clinical photographs. We have enclosed a leaflet providing more information about your consent for clinical photographs. We have also included a consent form for you to complete prior to your appointment. Any questions in regards to the consent form please either contact the Contact Centre on **(01904) 726400** or speak to a member of staff at your appointment.

We will ask you to attend the medical photography department for photographs to be taken. Photographs are taken according to a set protocol. They are used for planning your treatment. You will be asked if you are comfortable to be photographed without any clothing garments on. If not, you will be provided with a modesty garment. The photographs will be taken by a female photographer. If you would like a chaperone present, please ask the photographer and they will arrange this for you.

The medical photography office is located at Junction 3, Floor 2.

We look forward to seeing you on the day.

| **Personal Details / Label** | **Next of Kin** |
| --- | --- |
| Title : Dr Mr Mrs Ms Miss  First Name:  Surname:  Date of birth:  Preferred name:  Address:  Home Tel. No:  Occupation:  Work No:  Mobile No: | Name:  Relationship:  Address:  Home Tel. No:  Mobile No: |
| GP name:  GP surgery: | 2^nd^ Contact  Name:  Relationship to you?:  Tel. No: |

**Questionnaire:**

Please tick Yes or No to the following questions and give further details you think may be helpful to us.

| **1. Your Hernia:** | **Yes** | **No** | **Further details** |
| --- | --- | --- | --- |
| Does your hernia cause you problems? |  |  |  |
| Is it painful? |  |  |  |
| Do you ever have episodes of vomiting? |  |  |  |
| Have you ever had an operation(s) on your hernia before? |  |  |  |
| If ‘yes’ then please provide the following details for **each** of your previous hernia repairs: | | | |

| **First Hernia Repair:** | **Details** | | |
| --- | --- | --- | --- |
| In what year was this surgery performed? |  | | |
| Which hospital? |  | | |
| Which surgeon? |  | | |
|  | **Yes** | **No** | **Further Details** |
| Was the surgery performed laparoscopically i.e. by keyhole surgery? |  |  |  |
| Was a mesh used? |  |  |  |
| Did the wound on your tummy breakdown after surgery? |  |  |  |
| If ‘yes’ then how long did it take to finally heal? |  |  |  |

| **Second Hernia Repair:** | **Details** | | |
| --- | --- | --- | --- |
| In what year was this surgery performed? |  | | |
| Which hospital? |  | | |
| Which surgeon? |  | | |
|  | **Yes** | **No** | **Further Details** |
| Was the surgery performed laparoscopically i.e. by keyhole surgery? |  |  |  |
| Was a mesh used? |  |  |  |
| Did the wound on your tummy breakdown after surgery? |  |  |  |
| If ‘yes’ then how long did it take to finally heal? |  |  |  |

| **Third Hernia Repair:** | **Details** | | |
| --- | --- | --- | --- |
| In what year was this surgery performed? |  | | |
| Which hospital? |  | | |
| Which surgeon? |  | | |
|  | **Yes** | **No** | **Further Details** |
| Was the surgery performed laparoscopically i.e. by keyhole surgery? |  |  |  |
| Was a mesh used? |  |  |  |
| Did the wound on your tummy breakdown after surgery? |  |  |  |
| If ‘yes’ then how long did it take to finally heal? |  |  |  |

| **Fourth Hernia Repair:** | **Details** | | |
| --- | --- | --- | --- |
| In what year was this surgery performed? |  | | |
| Which hospital? |  | | |
| Which surgeon? |  | | |
|  | **Yes** | **No** | **Further Details** |
| Was the surgery performed laparoscopically i.e. by keyhole surgery? |  |  |  |
| Was a mesh used? |  |  |  |
| Did the wound on your tummy breakdown after surgery? |  |  |  |
| If ‘yes’ then how long did it take to finally heal? |  |  |  |

| **3. Previous Operations & Anaesthetics** |  |  |  |  | |
| --- | --- | --- | --- | --- | --- |
| Please give details of any operations that you have had?  **Operation:** | | | | **Hospital**  **And Surgeon** | **Year** |
|  | | | |  |  |
|  | **Yes** | **No** |  | **Further details** | |
| Have you ever had any problems with any previous anaesthetics? |  |  |  | If ‘yes’ please give details | |
| Have any of your relatives had problems with anaesthetics? |  |  |  | If ‘yes’ please give details | |

| **4. Body Weight** | **Yes** | **No** | **Further details** |
| --- | --- | --- | --- |
| Do you feel that you are overweight? |  |  |  |
| Have you tried to lose weight before? |  |  |  |
| What is the lowest weight you have been as an adult? | | | |
| What is the highest weight you have been as an adult? | | | |
| Is your weight: ☐ going up ☐ staying the same ☐ going down ☐ unsure | | | |

| **5. Activities / Exercise** | **Yes** | **No** | **Further details** |
| --- | --- | --- | --- |
| Are you working at the moment? |  |  |  |
| If ‘yes’ what kind of work do you do? | | | |
| How many times a week are you active for at least 30 minutes?  e.g. walking, swimming, gardening? | | | |
| Do you think that you could walk a mile? |  |  |  |
| Do you exercise regularly? |  |  |  |
| Do you use a mobility aid (e.g. sticks, walking frame or wheelchair)? |  |  |  |

| **6. Diabetes** | **Yes** | **No** | **Further details** |
| --- | --- | --- | --- |
| Do you have diabetes (diabetes mellitus)? |  |  |  |
| If ‘yes’ are you treated with insulin or tablets? |  |  |  |

| **7. Immunity** | **Yes** | **No** | **Further details** |
| --- | --- | --- | --- |
| Are you immunosuppressed? |  |  |  |
| Do you take steroids? |  |  |  |
| Have you ever been diagnosed as having any type of cancer? |  |  |  |

| **8. Smoking** | **Yes** | **No** | **Further details** |
| --- | --- | --- | --- |
| Do you smoke now? |  |  |  |
| If ‘yes’ would you like to give up? |  |  |  |
| If ‘no’ did you used to smoke? |  |  |  |
| If you used to smoke, when did you give up? | | | |
| How much did you used to smoke? | | | |

| **9. Infection** | **Yes** | **No** | **Further details** |
| --- | --- | --- | --- |
| Have you had any abdominal wound infections in the past? |  |  |  |
| If ‘yes’ please give details | | | |
| Have you ever suffered a serious infection (e.g. MRSA, clostridium difficile? |  |  |  |
| If ‘yes’ please give details | | | |
| Do you currently have a stoma? |  |  |  |
| Do you currently have a bowel fistula? |  |  |  |
| Do you currently have any open wounds / ulcers / blisters? |  |  |  |

| **10. Breathing Disorders** | **Yes** | **No** | **Further details** |
| --- | --- | --- | --- |
| Do you have asthma, chronic obstructive airways disease or any other breathing disorder? |  |  |  |
| Do you have asthma attacks more than once each month? |  |  |  |
| Do you have sleep apnoea? |  |  |  |

| **11. Heart Disease** | **Yes** | **No** | **Further details** |
| --- | --- | --- | --- |
| Do you get chest pain or become breathless climbing two flights of stairs? |  |  |  |
| Do you suffer with angina? |  |  |  |
| Have you had a heart attack? If ‘yes’ please give year |  |  |  |
| Are you currently being treated for an irregular heart beat? |  |  |  |
| Have you ever been treated for heart failure? |  |  |  |
| Have you ever been told that you have a heart murmur? |  |  |  |
| Are you being treated for high blood pressure? |  |  |  |
| Do you have a pacemaker or implanted defibrillator? |  |  |  |
| Do you have any coronary stents? |  |  |  |

| 12. Hormone, renal, liver & bleeding disorders | | Yes | No | Further details | |
| --- | --- | --- | --- | --- | --- |
| Do you have thyroid disease? | |  |  |  | |
| Have you ever been diagnosed with kidney disease? | |  |  |  | |
| Have you ever been diagnosed as having hepatitis? | |  |  |  | |
| Do you drink more than 1.5 pints of beer or 3 shorts or a half bottle of wine per day most days? | |  |  |  | |
| Have you ever been diagnosed as having a blood clot in the leg (deep vein thrombosis) or in the lung (pulmonary embolism)? | |  |  |  | |
| Have you or any close relative, been diagnosed with an inherited blood disorder such as sickle cell disease, clotting or bleeding disorder? | |  |  |  | |
| **13. Brain, nerve & musculoskeletal disorders** | **Yes** | | **No** | | **Further details** |
| Have you been diagnosed as having epilepsy? |  | |  | |  |
| Do you suffer from fainting or blackouts? |  | |  | |  |
| Have you ever had a minor (TIA) or major stroke? |  | |  | |  |
| Have you been diagnosed as having arthritis? |  | |  | |  |
| Are you able to lie flat comfortably? |  | |  | |  |

| **14. Medications** |  |  |  | |
| --- | --- | --- | --- | --- |
| Are you currently taking any medications (prescribed, herbal, over the counter, recreational, vitamins or other)?  Please give details (IN CAPITALS) or attach GP list | | | | |
| Name of medicine | Dose | | | Freq. |
| 1 |  | | |  |
| 2 |  | | |  |
| 3 |  | | |  |
| 4 |  | | |  |
| 5 |  | | |  |
| 6 |  | | |  |
| 7 |  | | |  |
| 8 |  | | |  |
| 9 |  | | |  |
| 10 |  | | |  |
| 11 |  | | |  |
| 12 |  | | |  |
| 13 |  | | |  |
| 14 |  | | |  |
| 15 |  | | |  |
| 16 |  | | |  |

| **Please indicate if you are taking any of the following?** | **Yes** | **No** | **Further details** |
| --- | --- | --- | --- |
| Anticoagulant tablets (for example aspirin, dipyridamole, warfarin, clopidogrel, prasugrel, dabigatran, apixiban) |  |  |  |

| **15. Allergies** | **Yes** | **No** | **Further details** |
| --- | --- | --- | --- |
| Have you ever had a reaction to medicines or other substances (e.g. food/topical agents/latex/metal/other)? If ‘yes’ please give details. |  |  |  |

| **16. Other medical conditions** | **Yes** | **No** | **Further details** |
| --- | --- | --- | --- |
| Is there any other medical condition or problem, not previously mentioned, that you feel we should know about? |  |  |  |


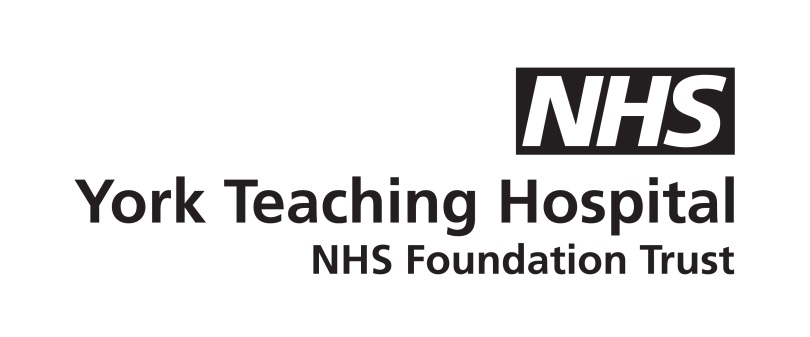
ABDOMINAL WALL RECONSTRUCTION

Clinical Assessment

| **Patient Name** | **DATE:**  **GI SURGEON:**  **PLASTIC SURGEON:**  **REFERRAL SOURCE:** |
| --- | --- |

| ***1. Body Dimensions:*** | ***Weight (kg)*** |  | ***Height (m)*** |  | ***BMI (kg/m2)*** |  |
| --- | --- | --- | --- | --- | --- | --- |

| **Skin Assessment:** | | **Anatomical Measurements (cm)** | |
| --- | --- | --- | --- |
| **Normal** | □ | **Xi / SP** |  |
| **Scarring** | □ | **ASIS /ASIS** |  |
| **Ulceration** | □ | **Divarication (cm)** |  |
| **Redundant Skin** | □ | **Rectus Tone** | |
| **Fistula** | □ | **Good** | □ |
| **Stoma** | □ | **Poor** | □ |

| 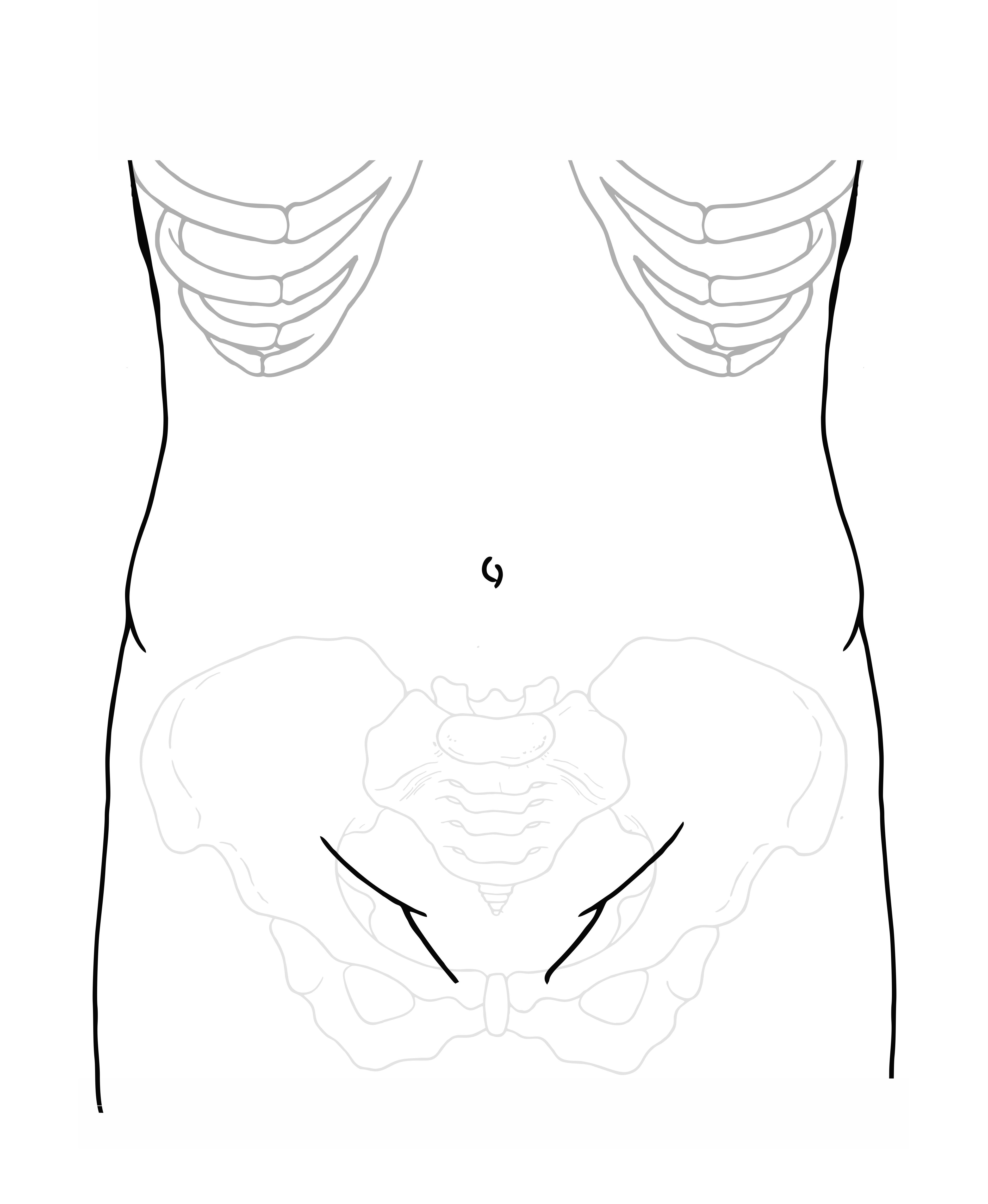 | **Hernia (s):** | **Length (cm)** | **Width (cm)** |
| --- | --- | --- | --- |
|  | **1** |  |  |
|  | **2** |  |  |
|  | **3** |  |  |
|  | **4** |  |  |
|  | **5** |  |  |
|  | **6** |  |  |

M and L Zones for Incisional Hernia

| Medial |  |  | Lateral |  |  |
| --- | --- | --- | --- | --- | --- |
| M1 | **Subxiphoidal** | xiphoid to 3 cm caudally | **L1** | **Subcostal** | between costal margin  and a horizontal line 3 cm  above umbilicus |
| M2 | **Epigastric** | 3 cm below xiphoid to 3 cm  above umbilicus | **L2** | **Flank** | lateral to rectal sheath  in area 3 cm above and  below umbilicus |
| M3 | **Umbilical** | 3 cm above to 3 cm below the  umbilicus | **L3** | **Iliac** | between a horizontal line 3  cm below umbilicus and  the inguinal region |
| M4 | **Infraumbilical** | 3 cm below umbilicus to 3 cm above pubis | **L4** | **Lumbar** | laterodorsal to anterior  axillary line |
| M5 | **Suprapubic** | pubic symphysis to 3 cm  cranially | **L5** |  |  |

VHWG Classification:

| Grade 1 | | Grade 2 | | Grade 3 | | Grade 4 | |
| --- | --- | --- | --- | --- | --- | --- | --- |
| *Low Risk* | | ***Comorbid*** | | ***Potentially Contaminated*** | | ***Infected*** | |
| □ | Low Risk for complications | □ | Smoker | □ | Previous wound infection | □ | Infected Mesh |
| □ | No history of wound infection | □ | Obese | □ | Stoma Present | □ | Septic Dehiscence |
|  |  | □ | Diabetic | □ | Violation of G.I.T. |  |  |
|  |  | □ | Immunosuppressed |  |  |  |  |
|  |  | □ | COPD |  |  |  |  |

Carolinas Equation for Determining Associated Risks (CeDar):

|  | Current Risk of Complications (%) |
| --- | --- |
|  | Target Weight to Reduce Complications (kg) |

Investigations:

|  | Investigation | Results: |
| --- | --- | --- |
| □ | **HbA1c** |  |
| □ | **MRSA Screen** |  |
| □ | **Wound(s)** |  |
| □ | **Spirometry** |  |
| □ | **CPX** | Anaerobic Threshold: |
| □ | **Endoscopy** | OGD:  Colonoscopy: |
| □ | **CT Scan** |  |

Clinical Photographs:

| □ | Photo Consent Form |
| --- | --- |
| □ | **Photo Info Booklet** |

Booklets:

| □ | Abdominal Wall Hernia (including STOPPA Mesh Repair and Component Separation technique) |
| --- | --- |
| □ | **Getting you Fitter for Complex Abdominal Wall Reconstruction Surgery** |
| □ | **Recovering from Major Abdominal Surgery** |

Pre-Operative Management:

**Optimisation:**

| □ | Weight | □ Tier 1:  □ Tier 2: Dietician Referral -> Sign Post  □ Tier 3: GP to Consider Referral  □ Tier 4: GP to Refer |
| --- | --- | --- |
| □ | **Exercise Regime** | □ GP to Refer to Exercise Programme  □ Advice in Clinic |
| □ | **Smoking Cessation** | □ GP to Refer  □ Advice in Clinic |
| □ | **Diabetic Management** | □ HbA1c  □ Endocrine Referral |
| □ | **Skin Care** | □ Moisturise  □ Dermatology Referral |
| □ | **Prehabilitation** | □ Calf Exercises Advice  □ Breathing Exercises Advice  □ Leaflet given |
| □ | **Abdominal Binder** | □ Issued in Clinic  □ Orthotic Referral |
| □ | **Pre-operative**  **Botulinum**  **Toxin** | □ Pre-op Botulinum Toxin |

Operative Plan:

### Surgeon(s):

| □ | General Surgeon | □ | Plastic Surgeon | □ | Other |
| --- | --- | --- | --- | --- | --- |

### Operative Duration:

| Number of Operating Sessions |  |
| --- | --- |
| Estimated Time (hr) |  |

**Planned Incisions:**

| 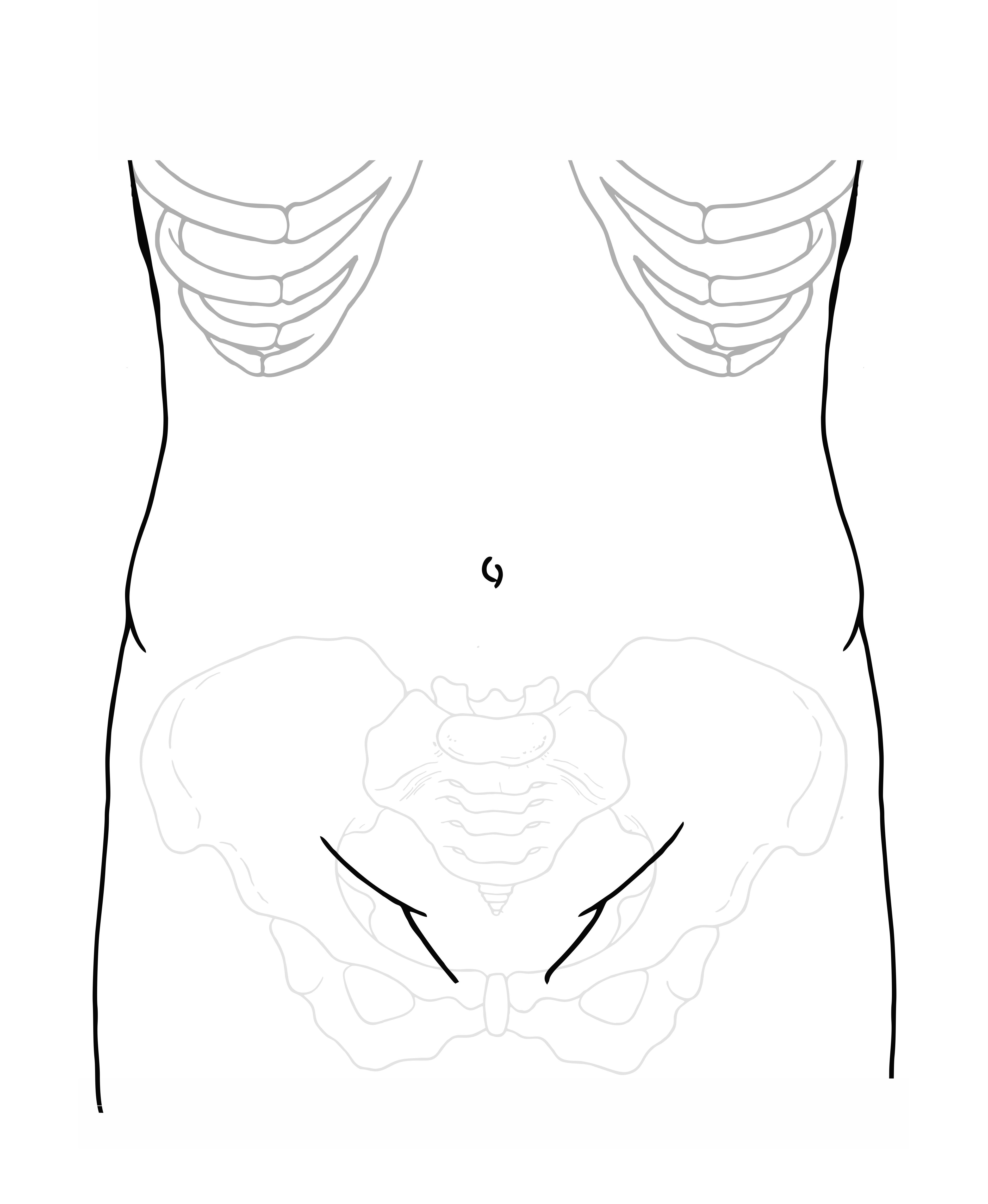 | **Stoppa Repair:** | | | | | | | | | |
| --- | --- | --- | --- | --- | --- | --- | --- | --- | --- | --- |
|  | □ | Retro rectus | □ | | Pre Peritoneal | | | □ | | TAR |
|  | **Component Separation:** | | | | | | | | | |
|  | **Right** | | | **Left:** | | | | | | |
|  | □ | Open | | □ | | Open | | | | |
|  | □ | Min Inv | | □ | | Min Inv | | | | |
|  | □ | None | | □ | | None | | | | |
|  | **Revision of Soft Tissues:** | | | | | | | | | |
|  | □ | Yes | | □ | | No | | | | |
|  | **Mesh:** | | | | | | | | | |
|  | □ | Synthetic | | □ | | Semi  synthetic | □ | | Biological | |
|  | Product: | | | | | | | | | |
|  | Size: | | | | | | | | | |
| **Additional Procedures:** |  | | | | | | | | | |
| **Bowel Prep:** |  | | | | | | | | | |

**COPY OF LETTER SENT TO PATIENT**

| **Current Episode:** | 1. Referred by 2. Seen in clinic today by |
| --- | --- |
| **Diagnosis:** |  |
| **Surgical History:** |  |
| **Hernia footprint:** | 1. Width -    cm 2. Height -   cm 3. Covers M 4. Covers L 5. VWHG Classification of Grade |
| **Smoker:** |  |
| **Diabetes:** |  |
| **BMI:** | 1. Height - 2. Weight - 3. BMI - |
| **CeDAR Calculation:** |  |
| **Target weight loss:** |  |
| **Plan:** |  |
| **Leaflets given:** | 1. Consent for photography 2. Complex abdominal wall hernia surgery 3. Recovering from major abdominal surgery |
| **Photography:** | 1. Sent to medical illustrations for photographs today |
| **Dietitian referral:** | Sent/Seen in clinic by |

To the patient:

This is a copy of a letter to health professionals which uses medical terms.  A copy is sent to you as a reminder of what was discussed in the clinic, so you know what has been passed on.  You may find it useful to keep copy letters and show them to other professionals when you meet them for the first time.  Please feel free to discuss anything in this letter when you next come to clinic.
